# Cold Weather Conditions and Risk of Hypothermia Among People Experiencing Homelessness: Implications for Prevention Strategies

**DOI:** 10.3390/ijerph16183259

**Published:** 2019-09-05

**Authors:** Paige Zhang, Kathryn Wiens, Ri Wang, Linh Luong, Donna Ansara, Stephanie Gower, Kate Bassil, Stephen W. Hwang

**Affiliations:** 1 MAP Centre for Urban Health Solutions, Li Ka Shing Knowledge Institute, 30 Bond Street, Toronto, ON M5B 1W8, Canada (P.Z.) (K.W.) (R.W.) (L.L.); 2 Dalla Lana School of Public Health, University of Toronto, 155 College St Room 500, Toronto, ON M5T 3M7, Canada (S.G.) (K.B.); 3 Toronto Public Health, 277 Victoria St, Toronto, ON M5B 1W2, Canada; 4 Division of General Internal Medicine, Department of Medicine, University of Toronto, Medical Sciences Building, 1 King’s College Cir, Toronto, ON M5S 1A8, Canada

**Keywords:** homelessness, meteorological conditions, hypothermia, cold weather policy

## Abstract

Hypothermia is a preventable condition that disproportionately affects individuals who experience homelessness, yet limited data exist to inform the response to cold weather. To fill this gap, we examined the association between meteorological conditions and the risk of hypothermia among homeless individuals. Hypothermic events were identified from emergency department charts and coroner’s records between 2004 and 2015 in Toronto, Canada. A time-stratified case-crossover design with conditional logistic regression was used to assess the relationship between the meteorological conditions (minimum temperature and precipitation) and the risk of hypothermia. There were 97 hypothermic events identified: 79 injuries and 18 deaths. The odds of experiencing a hypothermic event increased 1.64-fold (95% CI: 1.30–2.07) with every 5 °C decrease in the minimum daily temperature and 1.10-fold (95% CI: 1.03–1.17) with every 1 mm increase in precipitation. The risk of hypothermia among individuals experiencing homelessness increased with declining temperature; however, most cases occurred during periods of low and moderate cold stress. 72% occurred when the minimum daily temperatures were warmer than −15 °C. These findings highlight the importance of providing a seasonal cold weather response to prevent hypothermia, complemented by an alert-based response on extremely cold days.

## 1. Introduction

Homelessness continues to be a pressing social and public health concern, with over 100 million individuals estimated to be homeless worldwide [1]. Approximately 553,000 United States residents (17 per 10,000) and 35,000 Canadian residents (10 per 10,000) are homeless on any given night [2,3]. In Toronto, specifically, there were 8715 people who experienced homelessness on any given night in 2018 [4]. In many parts of North America, including Toronto, cold weather conditions prevail during the winter months. Individuals who experience homelessness often spend prolonged periods of time outside, which coupled with wet conditions, inadequate clothing, and mental health and substance use disorders could increase the risk of cold weather-related injury and death [5,6,7]. 

Hypothermia is a well-recognized consequence of exposure to cold environmental conditions, characterized by abnormally low body temperatures (<35 °C), cardiovascular, and central nervous system dysfunction, and potentially fatal outcomes [8,9]. Emergency department (ED) visits and hospital admissions for hypothermia could be very resource-intensive and often disproportionately represent individuals from disadvantaged groups [9,10]. For instance, individuals who experienced homelessness accounted for 1% of the ED visits, 24% of the hospitalizations, and 18% of the deaths attributed to hypothermia in New York City between 2005 and 2014 [11]. Further, individuals who were homeless in Poland had a nearly 13-times higher risk of death from hypothermia than individuals from the general population [12]. 

To reduce the risk of hypothermia among individuals who experience homelessness, organizations including public health units, shelter services, and street outreach programs often employ targeted approaches to cold weather planning and response. Cold weather alerts are typically issued during periods of extremely cold temperatures and are often linked to the opening of emergency warming facilities, provision of enhanced services by local agencies, and dissemination of information and advice on how to prevent hypothermia [13,14,15]. While many jurisdictions employ cold weather alerts, there is limited data on the links between meteorological conditions and health outcomes, especially for homeless populations [16]. To inform the cold-weather response, it is important to understand the association between cold weather conditions and health outcomes, such as hypothermia. Thus, the objective of this study was to examine the relationship between weather conditions and the risk of hypothermia among individuals experiencing homelessness in Toronto. 

## 2. Materials and Methods

### 2.1. Study Design

A time-stratified case-crossover design was used to analyze the relationship between the weather conditions and the risk of hypothermia among people experiencing homelessness in Toronto, Canada. The date of each hypothermic event was considered a case and up to four dates falling on the same weekday within the same month were chosen as controls. For example, if a hypothermic event occurred on February 22, 2012, then the corresponding control days would be February 1, 8, 15, and 29 of the same year. The use of a case-crossover design reduces the effect of potential confounders such as day of the week and seasonal effects [17]. 

### 2.2. Data Sources

#### 2.2.1. Hypothermic Events

A retrospective chart review was conducted to identify hypothermic events among people experiencing homelessness in Toronto, Ontario. Emergency department (ED) and coroner’s office records were reviewed for the period between 15 November 2004 and 31 March 2015.

The ED charts were reviewed at five Toronto hospitals: St. Michael’s Hospital, Mt. Sinai Hospital, Toronto General Hospital, Toronto Western Hospital, and St. Joseph’s Health Center. These hospitals were selected because of their proximity to the neighborhoods where there are high concentrations of people experiencing homelessness and organizations providing services for homeless people. Unpublished data from a previous study also indicated that these hospitals account for the large majority of emergency department visits by homeless persons in Toronto [18]. Hypothermic events were identified if the 10th version of the International Classification of Disease, Canada (ICD-10-CA) code for the most responsible diagnosis was hypothermia. Hypothermic events were excluded if the event occurred outside of the cold season (15 November to 31 March), another diagnosis was identified as the most responsible diagnosis, or the person experiencing the event was under the age of 18 years old. Charts were also excluded if the patient left before being seen or formally diagnosed by a physician.

Coroner’s records were reviewed to identify all hypothermic deaths in Toronto, using the ICD-10-CA code for hypothermia as the primary cause of death. A hypothermic event that was identified in both an ED record and the coroner’s records (i.e., when an individual presented to the ED with hypothermia and subsequently died) was counted as a single event.

Individuals were classified as experiencing homelessness at the time of the hypothermic event if the medical record or coroner’s record included at least one of the following:

1. Documentation of the individual’s residence at the time of the event or during the seven days preceding the event as being a shelter, public place, vehicle, abandoned building, or someone else’s home and not having their own place,
2. A clinical note stating the individual’s housing status as “homeless”, or
3. Documentation of the individual’s residential address as “no fixed address (NFA)” or the address of a shelter.

In cases of conflicting or uncertain housing information, clinical documentation took precedence over the residential address data. If an individual was not found to be homeless based on the above criteria, or if their housing status remained uncertain after a full review, the hypothermic event was excluded from further analysis. 

#### 2.2.2. Meteorological Variables

The climate in Toronto is classified as ‘dfb’ under the Köppen-Geiger climate classification, which is characterized by snow as the main climate (d), fully humid precipitation (f), and warm summer temperature (b) [19].

Meteorological data from the weather station at Toronto Pearson International Airport (World Meteorological Organization ID 71624), which is Toronto’s main weather station, were obtained online from Environment and Climate Change Canada. Data from this station are used by Toronto Public Health to determine when to issue extreme cold weather alerts. Meteorological data were examined as continuous variables for minimum daily temperature (°C), mean daily temperature (°C), minimum hourly wind chill, mean hourly wind chill, and total precipitation (mm) for each day during the study period. Minimum daily temperature was the lowest temperature reached in a 24-hour period. Mean daily temperature was the average of the maximum and minimum temperatures on each day. Wind chill, a temperature-like measure without units, was calculated using both temperature and wind velocity to account for the cooling effect of the wind [20]. Wind chill variables were calculated from 24 hourly measurements. If wind chill was missing for a given hour, the corresponding hourly temperature measurement was used in its place. Total precipitation (mm) is the sum of the rainfall or water equivalent (e.g., snow, freezing rain, hail) that falls to the ground each day. 

### 2.3. Statistical Analysis

Conditional logistic regression models were used to examine the link between meteorological variables and the risk of hypothermic events among individuals experiencing homelessness. Each set of cases and the corresponding control dates formed strata to fit into the regression model. Due to the highly related nature of the temperature and wind chill variables, only the minimum temperature and total precipitation were retained as independent variables in the logistic regression models. Potential non-linear effects of minimum temperature and precipitation were tested using a restricted cubic spline function in two univariate models. As no non-linear effects were detected, simple linear effects were reported in the final models. 

When comparing the models, the goodness of fit statistics (AIC, Hosmer-Lemeshow goodness of fit test) were similar regardless of whether the minimum temperature, mean temperature, minimum wind chill, or mean wind chill were modeled. The model with minimum temperature was deemed most relevant for public health action, as it is one of the factors used to issue extreme cold weather alerts in Toronto (along with wind chill and consideration of sudden shifts in temperature) and is readily understood and available to decision-makers and stakeholders through local weather forecasts. The predictive accuracy of the models was examined using the area under receiver operating characteristic curves (ROC).

Unadjusted and adjusted odds ratios and 95% confidence intervals (95% CI) were used to estimate the associations between the minimum temperature, precipitation, and the risk of hypothermia. The predicted odds ratio estimates were also presented for hypothermic injury or death at varying temperatures, with 0 °C as the reference temperature. In a separate model, the minimum temperature was replaced with the mean wind chill and the associated odds ratios are presented in a supplemental table for comparison. The primary analysis included all hypothermic events, regardless of whether it was a first occurrence or a repeated hypothermic event for a specific individual. To ensure that individuals who experienced multiple hypothermic events did not unduly affect the results, a sensitivity analysis was performed using only the first hypothermic event for each individual and excluding any subsequent hypothermic events. 

All statistical analyses were completed in R version 3.4.0 (Computing, Vienna, Austria) [21] and the conditional logistic models were fit using clogit from the survival package. 

### 2.4. Ethics Approval

The study was conducted in accordance with the Declaration of Helsinki, and the protocol was approved by the Ethics Committee of Toronto Public Health (REB 2015-06), Mount Sinai Hospital (REB 15-0300-C), St. Joseph’s Health Center (REB 16-801), and St. Michael’s Hospital (REB 14-403). We obtained a waiver of consent that complies with Canada’s Tri-council Policy Statement (TCPS2) [22], as the following criteria were met: (a) the research involves essentially no risk to the participants; (b) the lack of the participant’s consent is extremely unlikely to adversely affect the welfare of the participant; (c) it is impossible to carry out the research and to answer the research question properly, given the research design, if the prior consent of the participant is required; (d) it is not possible to debrief participants at a later time during the study; (e) the research does not involve any intervention. The study was also approved under a research agreement between the Office of the Chief Coroner and St. Michael’s Hospital.

## 3. Results

**Figure 1:**
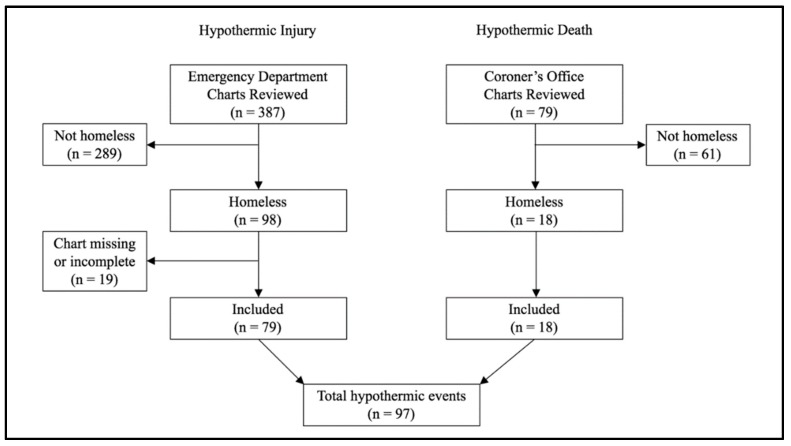
Flow chart describing the inclusion criteria for the hypothermic events identified from a review of the emergency department charts and Coroner’s office reports in Toronto (*n* = 97).

A total of 387 ED visits for hypothermia were identified, of which 98 (25%) were visits by individuals who met the criteria for homelessness. Of these, 19 (19%) visits were excluded because the individuals left the ED without being seen or formally diagnosed by a physician, leaving 79 hypothermic injuries for analysis. A total of 79 deaths due to hypothermia were identified in the coroner’s records, of which 18 (23%) deaths occurred in individuals who were classified as homeless. Overall, 97 (22%) hypothermic events (ED visits and deaths) occurred among individuals who met the criteria for homelessness (Figure 1).

The 97 hypothermic events were experienced by 87 individuals, of whom most were males (85%) and between the ages of 35 and 54 years (56%). Table 1 reports the characteristics of the hypothermic events included in the study. Of the 79 hypothermic injuries identified with ED charts, 25% resulted in an admission to the hospital. More than half of all hypothermic events occurred in either December (30%) or January (31%), and 72% of the hypothermic events occurred during low to moderate cold stress when minimum temperatures were warmer than −15 °C.

Table 2 reports the unadjusted and adjusted odds ratios for every 5 °C decrease in minimum daily temperature or a 1 mm increase in precipitation. The adjusted model indicates that the odds of a hypothermic event increased by 1.64-fold for every 5 °C decrease in the minimum temperature (95% CI: 1.30–2.07) and 1.10-fold for every 1 mm increase in precipitation (95% CI: 1.03–1.17). When compared to 0 °C, the predicted odds of a hypothermic event were 2.68 (95% CI: 1.68–4.27) times higher at −10 °C, 4.39 (95% CI: 2.18–8.84) times higher at −15 °C, and 7.18 (95% CI: 2.82–18.27) times higher at −20 °C (Table 3). The Hosmer-Lemeshow statistics showed a good model fit (*p* = 0.83); however, the area under the ROC curve was relatively low (0.69).

For comparison, the odds ratios for mean wind chill are reported in Supplementary Table S1. The adjusted results were similar to minimum temperature, although the magnitude of the association for wind chill was slightly weaker (OR = 1.49; 95% CI: 1.25–1.77). The results did not change when restricted to the first occurrence of hypothermia in participants with repeated events.

## 4. Discussion

Individuals who experience homelessness often spend prolonged periods of time outside, which makes them particularly susceptible to the detrimental effects of extreme cold weather conditions. To inform cold weather responses that target the needs of homeless individuals in large urban cities, our study examined the association between meteorological conditions and hypothermic injury or death among individuals who are homeless in Toronto. The results showed that 25% of all hypothermic injuries and 20% of hypothermic deaths were attributed to individuals experiencing homelessness. A recent study in New York reported a similar proportion of deaths (18%) and hospital admissions (24%) to occur among individuals who were homeless; yet, only 1% of ED visits were attributed to homeless individuals [11]. 

Another important finding is that nearly 20% of homeless individuals who presented with hypothermia left the ED without being seen or formally diagnosed by a physician. Similarly, Brown et al. reported 17% of homeless individuals who visited the ED left without seeing a physician during cold weather conditions in England [5]. For comparison, in the general Ontario population, the median annual rate of those who left the ED without being seen was 3.6% between 2003 and 2008 [23]. Previous research reports that individuals from lower socioeconomic groups are more likely to leave the ED without being seen [24], and lengthy wait times have been cited as one of the main reasons for abandoning care-seeking [23,24]. While our study did not examine the mean wait times, it would be beneficial for future work to compare ED wait times between homeless and non-homeless individuals presenting with hypothermia to inform the health system response to homeless individuals during the cold-weather season. 

We also observed that the odds of hypothermia increased by 1.64-fold (95% CI: 1.30–2.07) with every 5 °C decrease in the minimum daily temperature and 1.10-fold (95% CI: 1.03–1.17) with every 1 mm increase in precipitation. In Toronto, extreme cold weather alerts are issued when the forecasted minimum temperature is −15 °C or colder, or when the minimum wind chill is −20 or colder [25]. Yet, our results indicate that the odds of a hypothermic event were already 4.39-fold (95% CI: 2.18–8.84) higher at −15 °C compared to 0 °C. Notably, 72% of the hypothermic events occurred when the minimum daily temperature was warmer than −15 °C. This finding is consistent with previous reports that cold weather injuries occur in the general population during low (>0 °C) and moderate (0−13°C) cold stress, in addition to high-cold stress [6,9]. In Toronto, the average daily minimum temperatures typically range from −9 °C to −6 °C in December, January, and February [26]. While the highest relative risk of hypothermia was observed on the coldest days, over the course of a typical Toronto cold weather season, there are far more moderately cold days than extremely cold days. The lower relative risk on each moderate cold day accumulates over the course of the winter resulting in more cold-related health impacts at the population level. The general winter weather pattern of a smaller number of extremely cold days compared with more moderately cold days would be similar for many winter cities, suggesting that agencies involved in planning the cold weather response should routinely consider the health impacts associated with moderate cold stress along with those arising from extreme temperatures. In particular, it is important for local agencies involved in cold weather response to emphasize strategies to prevent hypothermia for the duration of the winter season, not only on extremely cold days. Further, the imperfect predictive ability of our model (the area under the ROC curve was 0.69) suggests that there were unmeasured factors beyond temperature and precipitation that likely contributed to the risk of hypothermia among individuals who experienced homelessness. These findings further support the need for a seasonal cold-weather response. Currently in Toronto, the cold weather response approach includes seasonal elements, such as 24-hour respite centres available throughout the winter, as well as shelter and warming centres that are made available during extremely cold weather alerts [27,28]. Future work should examine the impact of this joint approach on reducing the risk of hypothermia among individuals experiencing homelessness.

Our study has limitations to note. First, while all deaths attributed to hypothermia were captured from provincial coroner’s data, ED records from only select hospitals were reviewed. These hospitals serve the majority of the homeless population in Toronto; however, homeless individuals who presented with hypothermia at other hospitals would have been missed. This is expected to be a relatively small number of cases, given the concentration of individuals who experience homelessness in the downtown core. Second, ED visits for hypothermia were identified on the basis of the main diagnosis, and it is possible that visits for hypothermia documented as a secondary diagnosis were missed. Third, since there is no standard process of recording homelessness in the ED or coroner’s records, it is also possible that individuals who experienced homelessness were not identified due to a lack of documentation. Fourth, 19% of homeless individuals who presented with apparent hypothermia left the ED without being seen or formally diagnosed by a physician. Collectively, these limitations contribute to our study under-reporting the number of hypothermic events experienced by homeless individuals. Nonetheless, this under-reporting is expected to occur independently of meteorological conditions. A final consideration is that meteorological data were taken from a single monitoring site in Toronto, and these data may not represent the exact weather conditions experienced by each individual due to variations in temperature, wind chill, and precipitation across the city. 

## 5. Conclusions

Hypothermia is a potentially lethal but highly preventable condition. As such, it is essential for public health, shelter, and outreach organizations to take proactive steps through planning and response to mitigate the health impacts of cold weather on people experiencing homelessness. Since hypothermic events occur during periods of low and moderate cold stress in addition to high cold stress, a multifaceted cold weather response is required. This study provided a health-based rationale to inform the evolution of a local approach to cold weather response, and highlighted the critical importance of a seasonal cold weather response strategy comprised of low-barrier access to seasonal shelters and warming centers in addition to extreme cold weather alerts. Future work should examine the impact of this joint approach on reducing the risk of hypothermia among individuals experiencing homelessness. In the meantime, the ultimate public health goal must remain the elimination of homelessness through policy change and increased availability of affordable housing and appropriate supports.

## Figures and Tables

**Table 1:** Characteristics of the hypothermic events among the homeless population in Toronto from 15 November 2004 to 31 March 2015 (*n* = 97).

Characteristic	*n* (%)
Type of Event	
Hypothermic Injury	79 (81.4%)
Hypothermic Death	18 (18.6%)
Hospital Admission ^a^	
Yes	20 (25.3%)
No	59 (74.7%)
Month of Event	
November	14 (14.4%)
December	29 (29.9%)
January	30 (30.9%)
February	15 (15.5%)
March	9 (9.3%)
Minimum Daily Temperature (°C)	
>0	8 (8.3%)
−4.9 to 0	18 (18.8%)
−9.9 to −5	25 (26.0%)
−14.9 to −10	18 (18.8%)
−15 or colder	27 (28.1%)
Mean Daily Temperature (°C) ^b^	
>0	21 (21.6%)
−4.9 to 0	24 (24.7%)
−9.9 to −5	23 (23.7%)
−10 or colder	29 (29.9%)
Minimum Hourly Wind Chill	
>0	11 (11.8%)
−9.9 to 0	15 (16.1%)
−19.9 to −10	32 (34.4%)
−20 or colder	35 (37.6%)
Mean Hourly Wind Chill	
>0	17 (17.5%)
−9.9 to 0	28 (28.9%)
−19.9 to −10	33 (34.0%)
−20 or colder	19 (19.6%)
Mean Daily Precipitation (mm) ^c^	
0	47 (49.0%)
>0 to 5	34 (35.4%)
>5 to 10	7 (7.3%)
>10	8 (8.3%)

^a^: Hospital admission following an emergency department visit (*n* = 79); ^b^: The mean daily temperature was capped at −10 °C or colder due to the small sample size at colder temperatures; ^c^: Precipitation data was missing for one date.

**Table 2:** The odds ratios for the association between a 5 °C drop in the minimum daily temperature and hypothermic injury or death (*n* = 97 events) ^a^.

Meteorological Variables	Unadjusted Odds Ratio (95% CI)	Adjusted Odds Ratio (95% CI) ^b,c^
Minimum Temperature (per 5 °C decrease)	1.50 (1.21–1.88)	1.64 (1.30–2.07)
Precipitation (mm) ^c^	1.07 (1.01–1.13)	1.10 (1.03–1.17)

^a^: Conditional logistic regression models; ^b^: Adjusted models include the minimum temperature and precipitation; ^c^: Models with precipitation have 96 events due to missing data at one event date.

**Table 3:** Predicted odds ratio estimates of hypothermic injury or death at varying temperatures relative to the reference temperature (0 °C)^.^

Minimum Temperature (°C)	Predicted Odds Ratio (95% CI) ^a^
5	0.61 (0.48–0.77)
0	Ref.
−5	1.64 (1.30–2.07)
−10	2.68 (1.68–4.27)
−15	4.39 (2.18–8.84)
−20	7.18 (2.82–18.27)

^a^: Derived from the conditional logistic regression model, adjusted for precipitation.

## Supplementary Materials

The following are available online at https://www.mdpi.com/1660-4601/16/18/3259/s1, Table S1: Odds ratios for the association between a 5 °C drop in mean hourly wind chill and hypothermic injury or death.
