# Supplementary material for: Cold Weather Conditions and Risk of Hypothermia Among People Experiencing Homelessness: Implications for Prevention Strategies
Source: Int J Environ Res Public Health. 2019 Sep 5;16(18):3259. doi: 10.3390/ijerph16183259 (PMC6765826; doi:10.3390/ijerph16183259)
Supplement: Supplementary file 1 [file ijerph-16-03259-s001.pdf]

International Journal of
Environmental Research and Public Health

MDPI

Supplementary Materials: Cold Weather Conditions and Risk of Hypothermia Among People Experiencing Homelessness: Implications for Prevention Strategies

Table S1. Odds ratios for the association between a 5 °C drop in the mean hourly wind chill and hypothermic injury or death (n = 97 events) [a].

**Meteorological Variables**	**Unadjusted Odds Ratio (95% CI)**	**Adjusted Odds Ratio (95% CI)** [b, c]
Minimum Temperature (per 5 °C decrease)	1.41 (1.19–1.66)	1.49 (1.25–1.77)
Precipitation (mm) [c]	1.07 (1.01–1.13)	1.10 (1.03–1.17)

[a]: Conditional logistic regression models; [b]: Adjusted models include the mean hourly wind chill and precipitation; [c]: Models with precipitation have 96 events due to missing data at one event date.
